# Gluten-Free Diet Adherence Evaluation in Adults with Long-Standing Celiac Disease

**DOI:** 10.3390/foods14010076

**Published:** 2024-12-31

**Authors:** Marek K. Kowalski, Danuta Domżał-Magrowska, Piotr Szcześniak, Magdalena Bulska, Daria Orszulak-Michalak, Ewa Małecka-Wojciesko

**Affiliations:** 1 Department of Digestive Tract Diseases, Medical University of Lodz, 90-153 Lodz, Poland; mekamed14@gmail.com (M.K.K.); danuta.magrowska@gmail.com (D.D.-M.); 2 Department of Biopharmacy, Medical University of Lodz, 90-151 Lodz, Poland; piotr.szczesniak@umed.lodz.pl (P.S.); magdalena.bulska@umed.lodz.pl (M.B.); daria.orszulak-michalak@umed.lodz.pl (D.O.-M.)

**Keywords:** celiac disease, gluten-free diet, gluten-free diet adherence

## Abstract

Background: Celiac disease (CD) is an autoimmune disease that results from the interaction of genetic, immune, and environmental factors. According to the 2020 European Society for Pediatric Gastroenterology Hepatology and Nutrition (ESPGHAN) guidelines, an elimination diet (i.e., excluding products that may contain gluten) is the basic method of treating celiac disease. Following a gluten-free diet is extremely problematic, and patients often make unconscious deviations from the diet. According to the current Oslo definitions for celiac disease, depending on the clinical picture and adequate tests, several forms of celiac disease have been identified: typical, atypical, asymptomatic, potential, and refractory. Objective: The aim of the study was to assess the frequency of conscious diet mistakes and unconscious deviations from a gluten-free diet in a group of patients with long-standing celiac disease and their impact on the frequency of typical and atypical symptoms. Methods: The study included 57 people diagnosed with celiac disease between 1980 and 2010. After verifying the history of the disease according to the ESPGHAN guidelines from 2020, we excluded 19 patients who had Marsh grade 1 at the time of diagnosis or those without HLA DQ2 or DQ8 haplotypes detected. After verification, the study included 38 patients, 30 women and 8 men, with a verified diagnosis of typical celiac disease. The effectiveness of the gluten-free diet was assessed in all participants. Blood was collected to determine IgA anti-tissue transglutaminase II antibodies (anti-tTG) and IgG antibodies against deamidated gliadin peptides by ELISA. All survey participants provided data concerning current gastrointestinal and systemic symptoms, bowel habits, comorbidities, dietary habits, physical activity, and socioeconomic conditions. Results: A total of 25 patients (65.78%) declared strict adherence to the gluten-free diet. However, in this group, seven (18.4%) patients had significantly increased levels of anti-tTG antibodies (mean 82.3 RU/mL ± 78.9 SD at N < 20 RU/mL). Among the patients who consciously made dietary mistakes, six (46.2%) demonstrated increased levels of anti-tTG antibodies. The analysis did not reveal any difference between the frequency of intestinal and extraintestinal symptoms in patients making dietary mistakes and following the gluten-free diet. Conclusions: More than half of celiac patients unconsciously or consciously make dietary mistakes, which indicates an urgent need to increase their general knowledge of CD and the appropriate diet. Regardless of whether the gluten-free diet is followed, both typical and atypical symptoms of the disease have been observed among celiac patients.

## 1. Introduction

Celiac disease is an autoimmune disease that results from the interaction of genetic, immune, and environmental factors. It is caused by an immune reaction induced by gluten and prolamin derivatives in genetically predisposed individuals with specific histocompatibility antigens (HLA-DQ2 or HLA-DQ8). After consuming products made from cereals rich in gluten, they are gradually digested in the stomach, duodenum, and small intestine. Contact between gluten degradation products and the small intestinal mucosa leads to a complex immune response and, at a later stage, to the production in the submucosa of characteristic antibodies against gliadin, deamidated gliadin peptides, endomysium, and tissue transglutaminase, which damage enterocytes and their stroma. This reaction leads to further morphological changes in the duodenal mucosa. Initially, this leads to hyperplasia of the intestinal crypts, and then to gradual atrophy of the intestinal villi.

Characteristic intestinal symptoms of celiac disease are diarrhea (13–96%), abdominal pain (8–90%), vomiting (26–33%), flatulence (5–10%), and fatty, foul-smelling stools [1,2,3,4]. Extraintestinal symptoms may be related to gastrointestinal distress, mainly due to malabsorption leading to numerous disorders affecting most systems. The most common are weight loss (44–60%), growth retardation in children (19–31%), and anemia (3–30%), including anemia due to iron (40%), folic acid (20%), and vitamin B_12_ deficiency (17%). Deficiency in fat-soluble vitamins A, D, E, and K is more common in celiac individuals than in the general population. As a result, osteopenia (54%) and osteoporosis (12%) are observed, mainly due to vitamin D deficiency (34%), as well as hypocalcemia leading to tetany [5,6,7,8,9]. Lowered bone mass in children is connected to low bone mineral density, vitamin D deficiency [10], and decreased calcium levels [5]. In adults, the main reason for osteopenia and osteoporosis is insufficient intake of calcium and inadequate diet [11,12]. In the severe stage of the disease, which is currently extremely rare, features of malnutrition, sarcopenia, IgA deficiency, total protein deficiency, hypoalbuminemia, peripheral edema, and ascites are observed [1,2,9,13,14,15]. In this group of patients, oral cavity pathologies were more frequently confirmed, primarily dental enamel defects, caries, and recurrent aphthous lesions [16,17,18,19,20,21]. Celiac women have a higher incidence of menstrual cycle disorders (absent, late, or irregular menstruation), infertility, multiple miscarriages, intrauterine growth restriction, and low-birth-weight children than the general population [22,23,24]. These patients, especially those untreated, develop numerous neurological disorders such as gluten ataxia, progressive cerebellar ataxia, spinocerebellar degeneration, epilepsy, restless legs syndrome, myopathy, and peripheral neuropathy associated with vitamin B1 and B12 deficiency, as well as dementia [25,26,27,28].

According to the 2012 and 2020 ESPGHAN guidelines, an elimination diet (i.e., excluding products that may contain gluten) is the basic method of treating celiac disease [29,30]. Consuming 10 mg of gluten daily in patients with celiac disease should not cause disease exacerbation, although the daily dose in some cases may be many times higher [31,32,33,34]. Certification standards allow for a gluten content of 20 ppm (20 mg/kg of product) in gluten-free products and 100 ppm in low-gluten products [35]. However, in most cases, constant adherence to a gluten-free diet, especially among young patients, leads to full recovery of the villi and resolution of the inflammatory infiltrate, despite the presence of trace amounts of gluten contamination in food [36]. In adults, especially those over the age of 60, histopathological changes may not undergo complete remission despite strict adherence to a gluten-free diet [37,38].

Due to the numerous difficulties that celiac patients encounter on a daily basis, it is necessary to take into account not only fully conscious and intentional deviations from the gluten-free diet but also errors resulting from product contamination, for example, in restaurants. Inadequate information on gluten-free products may also be considered.

Due to difficulties in adhering to a gluten-free diet, many new therapeutic techniques have been developed. Advanced research is being conducted on enzyme supplementation to facilitate digestion and thereby reduce gluten immunogenicity [39,40,41,42,43]. In addition, studies are being conducted on substances that repair the tight junction between enterocytes, eliminating the zonulin effect that increases intestinal permeability [44]. Numerous authors are investigating immune response modification in patients with celiac disease not responding to a gluten-free diet (NRCD) as well as refractory celiac disease (RCD), in particular [45,46,47,48,49,50,51,52]. Another approach is to inhibit tissue transglutaminase using various proteins developed for this purpose [53,54,55,56].

## 2. Purpose of the Study

According to the pathophysiology of celiac disease, enterocytes are damaged during its course, leading to gradual villous atrophy and crypt hyperplasia. In patients diagnosed with celiac disease, adherence to a gluten-free diet leads to the regeneration of intestinal villi and to normalization of the level of characteristic antibodies. Over the years, public awareness of the gluten-free diet and the availability of products have improved significantly. Therefore, it is necessary to evaluate the current effectiveness of the diet. To exclude regular, unconscious dietary errors, detailed interviews should be conducted and the concentration of anti-transglutaminase antibodies should be assessed. Following a gluten-free diet is extremely problematic and patients often make unconscious deviations from the diet. Identifying common dietary mistakes may support the need for further research into new methods of reducing immunogenic gliadin concentrations.

## 3. Aim

1. Assessment of the frequency of conscious dietary mistakes in a group of patients with long-standing celiac disease.
2. Assessment of the frequency of unconscious deviations from the gluten-free diet in patients with celiac disease.
3. Assessment of the impact of conscious and unconscious deviations from the gluten-free diet on the frequency of typical and atypical symptoms

## 4. Material and Methods

### 4.1. Study and Control Groups

The study included 57 people diagnosed with celiac disease between 1980 and 2010. The study was conducted from 2012 to 2014. After verifying the history of the disease according to the ESPGHAN guidelines from 2020, we excluded 19 patients who had Marsh grade 1 at the time of diagnosis or lacked HLA DQ2 or DQ8 haplotypes. Further examination included 38 patients, 30 women and 8 men, with a verified diagnosis of celiac disease. The patients included in the study were between 18 and 60 years old. The mean age of patients in the study group was 35.87 ± 10.74 years. At the time of diagnosis, the patients included in the study had symptoms suggestive of typical celiac disease and were confirmed to have Marsh grade 3 on histological analysis of duodenal mucosa samples. In addition, they were confirmed to have HLA-DQ2 or DQ8 genes.

### 4.2. Antibody Concentration in Patients Diagnosed with Celiac Disease

The effectiveness of the gluten-free diet was verified by measurement of a specific antibody (anti-tTG) in all participants. Blood was collected to determine IgA anti-tissue transglutaminase II antibodies and IgG antibodies against deamidated gliadin peptides with ELISA (IBL International GMBH, Hamburg, Germany). Enzyme-linked immunosorbent assay (ELISA) was performed using the sandwich ELISA technique. At the beginning, specific antibodies attached to a solid phase in the wells bind with the appropriate antigen. After washing away unbound antigens, the material to be tested can be added. In the next stage, tested antibodies are detected by a secondary enzyme-conjugated antibody (E-Ab) specific for human IgG. After the substrate reaction, the intensity of the color developed is proportional to the amount of IgG-specific antibodies detected. The data obtained are quantitatively evaluated with a standard curve using a conventional ELISA evaluation program. A good fit is provided with a 4-parameter function or spline approximation. The sample concentrations can be read directly from the standard curve.

### 4.3. Survey Study

All survey participants provided data concerning current gastrointestinal and systemic symptoms, bowel habits, comorbidities, dietary habits, physical activity, and socioeconomic conditions. Furthermore, the duration of the gluten-free diet, adherence, and the frequency of deviations (conscious diet mistakes) were assessed based on patient declarations.

### 4.4. Statistical Analysis

In the analyzed study, a group that strictly adhered to a gluten-free diet was compared to a group that consciously and unconsciously made dietary mistakes. Nominal variables are presented as percentages. To compare two nominal variables, the Chi^2^ test with Yates’ correction or Fisher’s exact two-tailed test was used, depending on the size of the study groups. Continuous variables were tested for normality of distribution using the Shapiro–Wilk test. In the case of a normal distribution, variables are presented as means and standard deviations. The level of statistical significance was *p* < 0.05.

Statistical analyses were performed using Statistica 10 (Statsoft, Tulsa, OK, USA).

## 5. Results

### 5.1. Diet Adherence in Examined CD Patients

**Figure 1:**
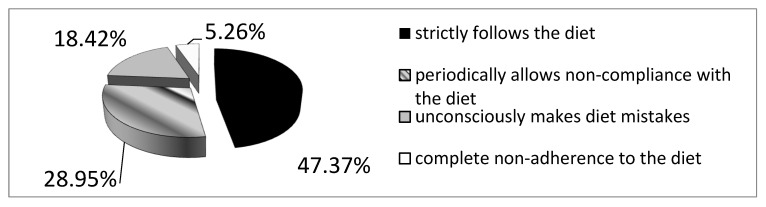
Declared adherence to the gluten-free diet in the group.

In the analyzed group of patients with CD, 25 patients (65.78%) declared strict adherence to the gluten-free diet. However, in this group, seven (18.4%) patients had significantly increased levels of anti-tTG antibodies (mean 82.3 RU/mL ± 78.9 SD at N < 20 RU/mL). After taking into account the group of patients who unconsciously made dietary mistakes, strict adherence to the gluten-free diet was confirmed in 47.4% of the study group (n = 18). Of the 13 patients declaring dietary deviations, two did not follow the gluten-free diet at all (Figure 1). The gender of the study participants did not influence the frequency of conscious and unconscious deviations from the gluten-free diet (Table 1).

### 5.2. Analysis of Anti-Tissue Transglutaminase Antibody Levels and Their Impact on Reported Symptoms

**Figure 2:**
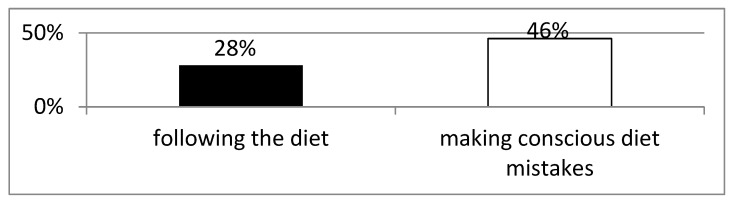
Evaluation of the frequency of elevated levels of anti-tissue transglutaminase antibodies (>20 UI/mL).

Among the patients who consciously made dietary mistakes, six (46.2%) demonstrated increased levels of anti-tTG antibodies (Figure 2). The mean duration from the CD, measured in months, was shorter in the group of patients who periodically deviated from the diet than in the group following a strict diet (49.8 months ± 55.6 SD vs. 73.5 months ± 99.1 SD).

**Figure 3:**
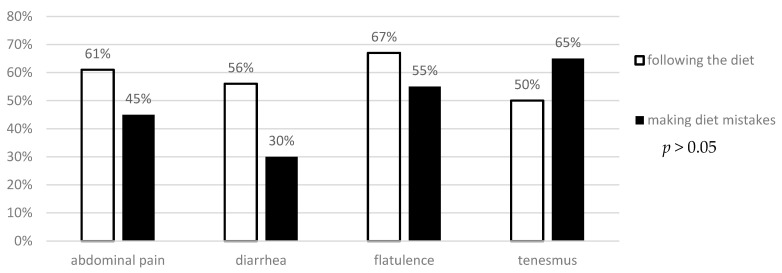
Evaluation of the frequency of intestinal symptoms in patients with celiac disease depending on gluten-free diet adherence.

**Figure 4:**
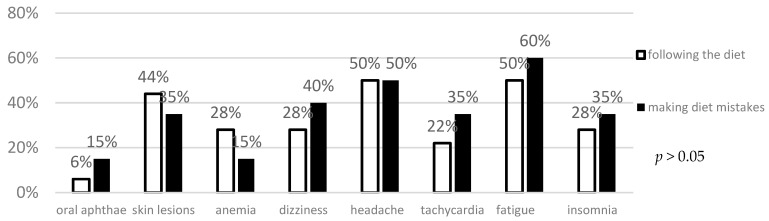
Evaluation of the frequency of extraintestinal symptoms in celiac patients depending on gluten-free diet adherence.

The analysis did not reveal any difference between the frequency of intestinal and extraintestinal symptoms in patients making dietary mistakes and following the gluten-free diet (Figure 3 and Figure 4).

## 6. Discussion

In the study group, only 65.78% of patients declared strict adherence to a gluten-free diet, 31.6% declared occasional deviations from the diet, whereas 5.26% did not follow the diet at all, mainly due to doubts regarding the diagnosis as well as the inconvenience of having to avoid certain foods. Moreover, among patients declaring a strict gluten-free diet, 28% were suspected of making dietary mistakes due to the detected elevated level of anti-tTG antibodies. Unconscious mistakes can be explained by numerous product contaminations and incorrect or incomplete descriptions on food labels. According to recent research, even in naturally gluten-free products, trace amounts of gluten are detected due to contamination resulting from production processes. This problem concerns both domestic [57,58,59] and foreign [60,61,62,63] products. Even in products with the crossed grain symbol, the safe gluten concentration is often exceeded [57,64,65,66]. Manufacturers of naturally gluten-free products omit information on the packaging about the possibility of gluten contamination during production [67]. Patients often make unconscious dietary mistakes and make minor deviations from the diet, mainly during social gatherings and when eating meals in restaurants [68,69,70]. Improper education and celiac patients’ insufficient knowledge about the disease and the gluten-free diet play a major role in unconscious dietary mistakes. In a survey conducted among 82 patients who had been following the diet for at least 6 years, no one correctly identified the gluten content of all 17 foods, and only 30% identified at least 14 foods correctly [71]. Research points to insufficient knowledge as one of the leading factors responsible for poor adherence to the gluten-free diet. This problem concerns both patients and medical staff [72,73,74]. Moreover, a survey conducted on a group of 584 patients with celiac disease showed that less than 30% of the respondents consciously make dietary mistakes [75]. In numerous studies, the frequency of conscious dietary errors ranges from 5 to 42%, depending on the country and the age of the study group [69,76,77,78,79]. One method of reducing errors was to provide gluten-free products on prescription, but no increase in the frequency of mistakes was observed after their withdrawal [80]. In the analyzed group, the mean time of gluten-free diet adherence was shorter in the group making dietary errors than in the group following the recommendations (49.8 months ± 55.6 SD vs. 73.5 months ± 99.1 SD). Meanwhile, Kurpp et al. and Webb et al. showed that patients diagnosed with celiac disease in adolescence or adulthood were more likely to follow dietary recommendations than those diagnosed with celiac disease in their youth [81,82].

In the conducted study, the level of IgA anti-tTG antibodies was determined to assess compliance with a gluten-free diet. In the study group, elevated levels of the abovementioned antibodies were found in 34.2% of patients, including 28% of patients following the diet and 46.2% of patients deviating from it. In a study by Ferreira et al., 29% of patients who had been following a gluten-free diet for years also had high levels of anti-tissue transglutaminase antibodies [83]. In turn, Gładyś et al. demonstrated elevated levels of IgA anti-tTG despite normal duodenal biopsy results in 27.3% of the study participants. They confirmed the correlation between increased antibody levels and dietary errors in two tests [84]. In studies conducted in celiac patients after gluten challenge, a gradual, slow increase in antibodies was observed over many months or even years. This increase concerned both the level of anti-tTG and endomysial antibodies (anti-EMA) but occurred mainly in patients consuming significant amounts of gluten for a long time [85,86,87,88]. In some patients, no increase in antibody levels was observed despite withdrawal from the gluten-free diet [89]. Also, in the analyzed group, some of the patients reporting deviations from the diet did not develop anti-tTG again, which may be related either to short periods of deviation or, contrary to their declarations, insufficient gluten intake.

In a study by Bufler et al., high IgA anti-tTG titer correlated with the occurrence of dietary deviations [90]. Bannister et al. also demonstrated a direct correlation between low antibody titers and good adherence to a gluten-free diet [91]. Galli et al. observed a correlation between significant changes in the intestinal mucosa and high antibody levels. Moreover, reintroduction of the gluten-free diet was not only associated with their reduction but in the group of patients in whom no histopathological improvement was observed, persistent antibody positivity was confirmed [92]. Qureshi [93] also reached similar conclusions in his meta-analysis. On the other hand, the consumption of foods containing small amounts of gluten, according to Selby et al., does not affect the risk of long-term villous atrophy [94]. To sum up, some studies confirm the great importance of antibodies in monitoring the gluten-free diet [95], but there is considerable controversy in this respect [96]. A number of studies indicate the lack of full intestinal villous recovery and making dietary errors while at the same time normalizing the level of anti-tissue transglutaminase antibodies [97,98,99]. This may be due to IgA deficiency [100], as well as to reactivity against other biomarkers, such as deamidated gliadin peptides or specific tissue transglutaminase epitopes [101]. For this reason, new biomarkers are sought for effective monitoring of adherence to the gluten-free diet [102,103,104]. The high frequency of dietary errors in the study group, including unconscious mistakes, may result from significantly more difficult contact with dietitians in Poland and the short duration of doctor appointments. This may explain the patients’ uncertain and inadequate knowledge about their diet. Furthermore, the presence of gluten in food declared as gluten-free cannot be ruled out with 100% certainty due to the lack of actual legal consequences in the case of contaminated products.

Nowadays, when we encounter highly processed food on a daily basis, the gluten-free diet has become an extremely demanding form of treatment. Patients must have knowledge of how to properly read food labels and follow current product testing for gluten content at the same time, taking into account the proper balance of the diet in terms of vitamins, minerals, and fiber [105]. This reduces their quality of life [106]. Moreover, gluten-free products have lower protein and fiber content as well as nutritional value than their gluten-containing counterparts. Due to the consumption of large amounts of meat, the gluten-free diet is also rich in saturated fats [107,108,109,110]. As a result, a poorly balanced gluten-free diet may lead to amino acid and protein deficiency, vitamin deficiency (especially vitamins A, B1, B6, B12, and D), electrolyte deficiency (especially iron, zinc, calcium, and magnesium), and folic acid deficiency [111,112,113,114]. Moreover, gluten-free products are many times more expensive than their gluten-containing counterparts, and some patients will be forced to limit their food intake or choose products of lower quality, which in many cases may be an additional factor intensifying deficiencies [115,116,117].

In the analyzed groups, no correlation was found between the reported complaints and proper adherence to the gluten-free diet. The lack of correlation between improper diet and the presented symptoms may result from the need for some patients to consume large doses of gluten over a long period of time. It should also be noted that the lack of a typical exacerbation of symptoms immediately after gluten consumption makes it much more difficult to identify products that may have been gluten-contaminated. In a study by Leffler et al., patients with confirmed celiac disease received 7.5 g of gluten for 28 days. Only 10% of the study participants developed symptoms [118]. In a study by Pedoto et al., also only 3.6% of the study participants reported symptoms, whereas 40.6% made dietary mistakes [119]. However, in a study by Silvester et al., as many as 72% of respondents declared discomfort after gluten consumption, mainly in the form of abdominal pain (80%). This study did not correlate the relationship between the declared symptoms and the objective increase in antibody titer [68]. Other studies also provide inconsistent data regarding the association between non-adherence to a gluten-free diet and reported symptoms. (Table 2) Those analyses are particularly difficult in asymptomatic CD patients, which could be included in the aforementioned studies [120]. However, regardless of the reported symptoms, the latest reports indicate a significant reduction in the quality of life of patients with celiac disease who do not follow a gluten-free diet [121]. The above studies may indirectly indicate a questionable correlation between deviations from the gluten-free diet—especially when these deviations are short-lasting—and the presented symptoms. However, such deviations undoubtedly affect the patients’ quality of life.

## 7. Summary

Celiac disease is a common autoimmune disease of the digestive tract. Currently, the only available treatment method is a strict diet that completely excludes gluten. Due to difficulties in adherence to the diet, appropriate education is necessary among patients to improve the effectiveness of compliance with dietary recommendations. Further research and development of new treatment techniques should also be considered.

## 8. Conclusions

1. More than half of celiac patients unconsciously or consciously make dietary mistakes.
2. Regardless of whether the gluten-free diet is followed, both typical and atypical symptoms of the disease have been observed among celiac patients.
3. Despite making conscious dietary mistakes, not all patients with celiac disease diagnosed years ago will redevelop antibodies against tissue transglutaminase.

## Figures and Tables

**Table 1:** Evaluation of the frequency of non-adherence to the gluten-free diet by gender.

Adherence to Gluten-Free Diet	Female	Male
Strictly follows the diet	43.3%	62.5%
Unconsciously makes dietary mistakes	20%	12.5%
Conscious non-compliance	36.7%	25%

**Table 2:** The association between non-adherence to a gluten-free diet and reported symptoms.

Author	Year	Sample Size	Frequency of Gluten-Free Diet Adherence	Frequency of Symptoms Correlating with Dietary Deviations
Leffler et al. [118]	2012	20	n/a	10%
Pedoto et al. [119]	May 2018–June 2019	160	40.6%	3.7%
Silvester et al. [68]	December 2012–September 2015	105	74.3%	72%
Czaja-Bulsa et al. [122]	2016–2017	102	26%	0%
Muhammed et al. [123]	2004–2014	375	37%	60.4%

## Data Availability

The original contributions presented in this study are included in the article. Further inquiries can be directed to the corresponding author.

## References

[1] Garampazzi A., Rapa A., Mura S., Capelli A., Valori A., Boldorini R., Oderda G. (2007). Clinical pattern of celiac disease is still changing. J. Pediatr. Gastroenterol. Nutr..

[2] Dinler G., Atalay E., Kalayci A.G. (2009). Celiac disease in 87 children with typical and atypical symptoms in Black Sea region of Turkey. World J. Pediatr..

[3] Bottaro G., Failla P., Rotolo N., Sanfilippo G., Azzaro F., Spina M., Patane R. (1993). Changes in coeliac disease behaviour over the years. Acta Paediatr..

[4] Faulkner-Hogg K.B., Selby W.S., Loblay R.H. (1999). Dietary analysis in symptomatic patients with coeliac disease on a gluten-free diet: The role of trace amounts of gluten and non-gluten food intolerances. Scand. J. Gastroenterol..

[5] Kalayci A.G., Kansu A., Girgin N., Kucuk O., Aras G. (2001). Bone mineral density and importance of a gluten-free diet in patients with celiac disease in childhood. Pediatrics.

[6] Mautalen C., Gonzalez D., Mazure R., Vazquez H., Lorenzetti M.P., Maurino E., Niveloni S., Pedreira S., Smecuol E., Boerr L.A. (1997). Effect of treatment on bone mass, mineral metabolism, and body composition in untreated celiac disease patients. Am. J. Gastroenterol..

[7] Bai J.C., Gonzalez D., Mautalen C., Mazure R., Pedreira S., Vazquez H., Smecuol E., Siccardi A., Cataldi M., Niveloni S. (1997). Long-term effect of gluten restriction on bone mineral density of patients with coeliac disease. Aliment. Pharmacol. Ther..

[8] Ciacci C., Maurelli L., Klain M., Savino G., Salvatore M., Mazzacca G., Cirillo M. (1997). Effects of dietary treatment on bone mineral density in adults with celiac disease: Factors predicting response. Am. J. Gastroenterol..

[9] Zanchetta M.B., Longobardi V., Costa F., Longarini G., Mazure R.M., Moreno M.L., Vazquez H., Silveira F., Niveloni S., Smecuol E. (2016). Impaired Bone Microarchitecture Improves After One Year on Gluten-Free Diet: A Prospective Longitudinal HRpQCT Study in Women with Celiac Disease. J. Bone Miner. Res..

[10] Aydemir Y., Erdogan B., Türkeli A. (2021). Vitamin D deficiency negatively affects both the intestinal epithelial integrity and bone metabolism in children with Celiac disease. Clin. Res. Hepatol. Gastroenterol..

[11] Hoteit M., Chamas Z., Assaf S., Bouhairie M.M., Bahr A., Daccache R., Matar R., Hallal M., Hotayt S., Hotayt B. (2022). Nutritional status, nutrient imbalances, food-related behaviors and dietary supplements use among patients with celiac disease on a gluten free diet in Lebanon: A national cross-sectional study. F1000Research.

[12] Gessaroli M., Frazzoni L., Sikandar U., Bronzetti G., Pession A., Zagari R.M., Fuccio L., Forchielli M.L. (2023). Nutrient intakes in adult and pediatric coeliac disease patients on gluten-free diet: A systematic review and meta-analysis. Eur. J. Clin. Nutr..

[13] Catal F., Topal E., Ermistekin H., Yildirim Acar N., Sinanoglu M.S., Karabiber H., Selimoglu M.A. (2015). The hematologic manifestations of pediatric celiac disease at the time of diagnosis and efficiency of gluten-free diet. Turk. J. Med. Sci..

[14] Repo M., Lindfors K., Maki M., Huhtala H., Laurila K., Lahdeaho M.L., Saavalainen P., Kaukinen K., Kurppa K. (2016). Anemia and Iron Deficiency in Children with Potential Celiac Disease. J. Pediatr. Gastroenterol. Nutr..

[15] Schosler L., Christensen L.A., Hvas C.L. (2016). Symptoms and findings in adult-onset celiac disease in a historical Danish patient cohort. Scand. J. Gastroenterol..

[16] Chang M.S., Minaya M.T., Cheng J., Connor B.A., Lewis S.K., Green P.H. (2011). Double-blind randomized controlled trial of rifaximin for persistent symptoms in patients with celiac disease. Dig. Dis. Sci..

[17] Bucci P., Carile F., Sangianantoni A., D’Angio F., Santarelli A., Lo Muzio L. (2006). Oral aphthous ulcers and dental enamel defects in children with coeliac disease. Acta Paediatr..

[18] Costacurta M., Maturo P., Bartolino M., Docimo R. (2010). Oral manifestations of coeliac disease: A clinical-statistic study. Oral Implantol..

[19] Avsar A., Kalayci A.G. (2008). The presence and distribution of dental enamel defects and caries in children with celiac disease. Turk. J. Pediatr..

[20] Cantekin K., Arslan D., Delikan E. (2015). Presence and distribution of dental enamel defects, recurrent aphthous lesions and dental caries in children with celiac disease. Pak. J. Med. Sci..

[21] Acar S., Yetkiner A.A., Ersin N., Oncag O., Aydogdu S., Arikan C. (2012). Oral findings and salivary parameters in children with celiac disease: A preliminary study. Med. Princ. Pract..

[22] Martinelli D., Fortunato F., Tafuri S., Germinario C.A., Prato R. (2010). Reproductive life disorders in Italian celiac women. A case-control study. BMC Gastroenterol..

[23] Singh P., Arora S., Lal S., Strand T.A., Makharia G.K. (2016). Celiac Disease in Women with Infertility: A Meta-Analysis. J. Clin. Gastroenterol..

[24] Lasa J.S., Zubiaurre I., Soifer L.O. (2014). Risk of infertility in patients with celiac disease: A meta-analysis of observational studies. Arq. Gastroenterol..

[25] Weinstock L.B., Walters A.S., Mullin G.E., Duntley S.P. (2010). Celiac disease is associated with restless legs syndrome. Dig. Dis. Sci..

[26] Manchanda S., Davies C.R., Picchietti D. (2009). Celiac disease as a possible cause for low serum ferritin in patients with restless legs syndrome. Sleep Med..

[27] Rodrigo L., Hernandez-Lahoz C., Lauret E., Rodriguez-Pelaez M., Soucek M., Ciccocioppo R., Kruzliak P. (2016). Gluten ataxia is better classified as non-celiac gluten sensitivity than as celiac disease: A comparative clinical study. Immunol. Res..

[28] Isikay S., Hizli S., Coskun S., Yilmaz K. (2015). Increased Tissue Transglutaminase Levels Are Associated with Increased Epileptiform Activity in Electroencephalography Among Patients with Celiac Disease. Arq. Gastroenterol..

[29] Husby S., Koletzko S., Korponay-Szabo I.R., Mearin M.L., Phillips A., Shamir R., Troncone R., Giersiepen K., Branski D., Catassi C. (2012). European Society for Pediatric Gastroenterology, Hepatology, and Nutrition guidelines for the diagnosis of coeliac disease. J. Pediatr. Gastroenterol. Nutr. USA.

[30] Husby S., Koletzko S., Korponay-Szabó I., Kurppa K., Mearin M.L., Ribes-Koninckx C., Shamir R., Troncone R., Auricchio R., Castillejo G. (2020). European Society Paediatric Gastroenterology, Hepatology and Nutrition Guidelines for Diagnosing Coeliac Disease 2020. J. Pediatr. Gastroenterol. Nutr..

[31] Lahdeaho M.L., Maki M., Laurila K., Huhtala H., Kaukinen K. (2011). Small-bowel mucosal changes and antibody responses after low- and moderate-dose gluten challenge in celiac disease. BMC Gastroenterol..

[32] Catassi C., Fabiani E., Iacono G., D’Agate C., Francavilla R., Biagi F., Volta U., Accomando S., Picarelli A., De Vitis I. (2007). A prospective, double-blind, placebo-controlled trial to establish a safe gluten threshold for patients with celiac disease. Am. J. Clin. Nutr..

[33] Bruins M.J. (2013). The clinical response to gluten challenge: A review of the literature. Nutrients.

[34] Akobeng A.K., Thomas A.G. (2008). Systematic review: Tolerable amount of gluten for people with coeliac disease. Aliment. Pharmacol. Ther..

[35] Gibert A., Espadaler M., Angel Canela M., Sanchez A., Vaque C., Rafecas M. (2006). Consumption of gluten-free products: Should the threshold value for trace amounts of gluten be at 20, 100 or 200 p.p.m.?. Eur. J. Gastroenterol. Hepatol..

[36] Zanini B., Marullo M., Villanacci V., Salemme M., Lanzarotto F., Ricci C., Lanzini A. (2016). Persistent Intraepithelial Lymphocytosis in Celiac Patients Adhering to Gluten-Free Diet Is Not Abolished Despite a Gluten Contamination Elimination Diet. Nutrients.

[37] Lanzini A., Lanzarotto F., Villanacci V., Mora A., Bertolazzi S., Turini D., Carella G., Malagoli A., Ferrante G., Cesana B.M. (2009). Complete recovery of intestinal mucosa occurs very rarely in adult coeliac patients despite adherence to gluten-free diet. Aliment. Pharmacol. Ther..

[38] Tursi A., Brandimarte G., Giorgetti G.M., Elisei W., Inchingolo C.D., Monardo E., Aiello F. (2006). Endoscopic and histological findings in the duodenum of adults with celiac disease before and after changing to a gluten-free diet: A 2-year prospective study. Endoscopy.

[39] Pultz I.S., Hill M., Vitanza J.M., Wolf C., Saaby L., Liu T., Winkle P., Leffler D.A. (2021). Gluten Degradation, Pharmacokinetics, Safety, and Tolerability of TAK-062, an Engineered Enzyme to Treat Celiac Disease. Gastroenterology.

[40] Murray J.A., Syage J.A., Wu T.T., Dickason M.A., Ramos A.G., Van Dyke C., Horwath I., Lavin P.T., Mäki M., Hujoel I. (2022). Latiglutenase Protects the Mucosa and Attenuates Symptom Severity in Patients with Celiac Disease Exposed to a Gluten Challenge. Gastroenterology.

[41] Tye-Din J.A., Anderson R.P., Ffrench R.A., Brown G.J., Hodsman P., Siegel M., Botwick W., Shreeniwas R. (2010). The effects of ALV003 pre-digestion of gluten on immune response and symptoms in celiac disease in vivo. Clin. Immunol..

[42] Siegel M., Garber M.E., Spencer A.G., Botwick W., Kumar P., Williams R.N., Kozuka K., Shreeniwas R., Pratha V., Adelman D.C. (2012). Safety, tolerability, and activity of ALV003: Results from two phase 1 single, escalating-dose clinical trials. Dig. Dis. Sci..

[43] Lahdeaho M.L., Kaukinen K., Laurila K., Vuotikka P., Koivurova O.P., Karja-Lahdensuu T., Marcantonio A., Adelman D.C., Maki M. (2014). Glutenase ALV003 attenuates gluten-induced mucosal injury in patients with celiac disease. Gastroenterology.

[44] Biopharma 9 Meters Biopharma Announces Interim Analysis of Phase 3 Study of Larazotide for Celiac Disease Does Not Support Trial Continuation. (Press Release 21 June 2022) 2024. https://www.accesswire.com/705856/9-Meters-Biopharma-Announces-Interim-Analysis-of-Phase-3-Study-of-Larazotide-for-Celiac-Disease-Does-Not-Support-Trial-Continuation.

[45] Klemenak M., Dolinsek J., Langerholc T., Di Gioia D., Micetic-Turk D. (2015). Administration of Bifidobacterium breve Decreases the Production of TNF-alpha in Children with Celiac Disease. Dig. Dis. Sci..

[46] Olivares M., Laparra M., Sanz Y., Cinova J., De Palma G., Stepankova R., Kofronova O., Kverka M., Tuckova L. (2011). Influence of Bifidobacterium longum CECT 7347 and gliadin peptides on intestinal epithelial cell proteome. J. Agric. Food Chem. USA.

[47] Olivares M., Castillejo G., Varea V., Sanz Y. (2014). Double-blind, randomised, placebo-controlled intervention trial to evaluate the effects of Bifidobacterium longum CECT 7347 in children with newly diagnosed coeliac disease. Br. J. Nutr..

[48] Smecuol E., Hwang H.J., Sugai E., Corso L., Chernavsky A.C., Bellavite F.P., Gonzalez A., Vodanovich F., Moreno M.L., Vazquez H. (2013). Exploratory, randomized, double-blind, placebo-controlled study on the effects of Bifidobacterium infantis natren life start strain super strain in active celiac disease. J. Clin. Gastroenterol..

[49] Rubio-Tapia A., Murray J.A. (2010). Classification and management of refractory coeliac disease. Gut.

[50] Mukewar S.S., Sharma A., Rubio-Tapia A., Wu T.T., Jabri B., Murray J.A. (2017). Open-Capsule Budesonide for Refractory Celiac Disease. Am. J. Gastroenterol..

[51] Therrien A., Silvester J.A., Leonard M.M., Leffler D.A., Fasano A., Kelly C.P. (2021). Enteric-Release Budesonide May Be Useful in the Management of Non-Responsive Celiac Disease. Dig. Dis. Sci..

[52] Lähdeaho M.L., Scheinin M., Vuotikka P., Taavela J., Popp A., Laukkarinen J., Koffert J., Koivurova O.P., Pesu M., Kivelä L. (2019). Safety and efficacy of AMG 714 in adults with coeliac disease exposed to gluten challenge: A phase 2a, randomised, double-blind, placebo-controlled study. Lancet Gastroenterol. Hepatol..

[53] Schuppan D., Mäki M., Lundin K.E.A., Isola J., Friesing-Sosnik T., Taavela J., Popp A., Koskenpato J., Langhorst J., Hovde Ø. (2021). A Randomized Trial of a Transglutaminase 2 Inhibitor for Celiac Disease. N. Engl. J. Med..

[54] Dotsenko V., Tewes B., Hils M., Pasternack R., Isola J., Taavela J., Popp A., Sarin J., Huhtala H., Hiltunen P. (2024). Transcriptomic analysis of intestine following administration of a transglutaminase 2 inhibitor to prevent gluten-induced intestinal damage in celiac disease. Nat. Immunol..

[55] Rauhavirta T., Oittinen M., Kivisto R., Mannisto P.T., Garcia-Horsman J.A., Wang Z., Griffin M., Maki M., Kaukinen K., Lindfors K. (2013). Are transglutaminase 2 inhibitors able to reduce gliadin-induced toxicity related to celiac disease? A proof-of-concept study. J. Clin. Immunol..

[56] Sulic A.M., Kurppa K., Rauhavirta T., Kaukinen K., Lindfors K. (2015). Transglutaminase as a therapeutic target for celiac disease. Expert Opin. Ther. Targets.

[57] Daniewski W., Wojtasik A., Kunachowicz H. (2010). Gluten content in special dietary use gluten-free products and other food products. Rocz. Panstw. Zakl. Hig..

[58] Cichańska B.A. (2009). Problemy z rozróżnianiem żywności bezglutenowej. Pediatr. Współcz. Gastroenterol. Hepatol. Zyw. Dziecka.

[59] Wojtasik A., Daniewski W., Kunachowicz H. (2010). Zawartość glutenu (gliadyny) w wybranych produktach spożywczych. Bromatol. Chem. Toksykol..

[60] Koerner T.B., Cleroux C., Poirier C., Cantin I., La Vieille S., Hayward S., Dubois S. (2013). Gluten contamination of naturally gluten-free flours and starches used by Canadians with celiac disease. Food Addit. Contaminants. Part A Chem. Anal. Control. Expo. Risk Assess..

[61] Thompson T., Lee A.R., Grace T. (2010). Gluten contamination of grains, seeds, and flours in the United States: A pilot study. J. Am. Diet. Assoc..

[62] Miller K., McGough N., Urwin H. (2016). Catering Gluten-Free When Simultaneously Using Wheat Flour. J. Food Prot..

[63] Colgrave M.L., Goswami H., Byrne K., Blundell M., Howitt C.A., Tanner G.J. (2015). Proteomic profiling of 16 cereal grains and the application of targeted proteomics to detect wheat contamination. J. Proteome Res..

[64] Gibert A., Kruizinga A.G., Neuhold S., Houben G.F., Canela M.A., Fasano A., Catassi C. (2013). Might gluten traces in wheat substitutes pose a risk in patients with celiac disease? A population-based probabilistic approach to risk estimation. Am. J. Clin. Nutr..

[65] La Vieille S., Dubois S., Hayward S., Koerner T.B. (2014). Estimated levels of gluten incidentally present in a Canadian gluten-free diet. Nutrients.

[66] Lee H.J., Anderson Z., Ryu D. (2014). Gluten contamination in foods labeled as “gluten free” in the United States. J. Food Prot..

[67] Sharma G.M., Pereira M., Williams K.M. (2015). Gluten detection in foods available in the United States—A market survey. Food Chem..

[68] Silvester J.A., Graff L.A., Rigaux L., Walker J.R., Duerksen D.R. (2016). Symptomatic suspected gluten exposure is common among patients with coeliac disease on a gluten-free diet. Aliment. Pharmacol. Ther..

[69] Roma E., Roubani A., Kolia E., Panayiotou J., Zellos A., Syriopoulou V.P. (2010). Dietary compliance and life style of children with coeliac disease. J. Hum. Nutr. Diet..

[70] Black J.L., Orfila C. (2011). Impact of coeliac disease on dietary habits and quality of life. J. Hum. Nutr. Diet..

[71] Silvester J.A., Weiten D., Graff L.A., Walker J.R., Duerksen D.R. (2016). Is it gluten-free? Relationship between self-reported gluten-free diet adherence and knowledge of gluten content of foods. Nutrition.

[72] Villafuerte-Galvez J., Vanga R.R., Dennis M., Hansen J., Leffler D.A., Kelly C.P., Mukherjee R. (2015). Factors governing long-term adherence to a gluten-free diet in adult patients with coeliac disease. Aliment. Pharmacol. Ther..

[73] Zarkadas M., Dubois S., MacIsaac K., Cantin I., Rashid M., Roberts K.C., La Vieille S., Godefroy S., Pulido O.M. (2013). Living with coeliac disease and a gluten-free diet: A Canadian perspective. J. Hum. Nutr. Diet..

[74] Silvester J.A., Weiten D., Graff L.A., Walker J.R., Duerksen D.R. (2016). Living gluten-free: Adherence, knowledge, lifestyle adaptations and feelings towards a gluten-free diet. J. Hum. Nutr. Diet..

[75] Sdepanian V.L., de Morais M.B., Fagundes-Neto U. (2001). Celiac disease: Evaluation of compliance to gluten-free diet and knowledge of disease in patients registered at the Brazilian Celiac Association (ACA). Arq. Gastroenterol..

[76] Hopman E.G., Koopman H.M., Wit J.M., Mearin M.L. (2009). Dietary compliance and health-related quality of life in patients with coeliac disease. Eur. J. Gastroenterol. Hepatol..

[77] Hauser W., Gold J., Stein J., Caspary W.F., Stallmach A. (2006). Health-related quality of life in adult coeliac disease in Germany: Results of a national survey. Eur. J. Gastroenterol. Hepatol..

[78] Chauhan J.C., Kumar P., Dutta A.K., Basu S., Kumar A. (2010). Assessment of dietary compliance to gluten free diet and psychosocial problems in Indian children with celiac disease. Indian J. Pediatr..

[79] Rashid M., Cranney A., Zarkadas M., Graham I.D., Switzer C., Case S., Molloy M., Warren R.E., Burrows V., Butzner J.D. (2005). Celiac disease: Evaluation of the diagnosis and dietary compliance in Canadian children. Pediatrics.

[80] Crocker H., Lewis T., Violato M., Peters M. (2024). The affordability and obtainability of gluten-free foods for adults with coeliac disease following their withdrawal on prescription in England: A qualitative study. J. Hum. Nutr. Diet..

[81] Kurppa K., Lauronen O., Collin P., Ukkola A., Laurila K., Huhtala H., Maki M., Kaukinen K. (2012). Factors associated with dietary adherence in celiac disease: A nationwide study. Digestion.

[82] Webb C., Myleus A., Norstrom F., Hammarroth S., Hogberg L., Lagerqvist C., Rosen A., Sandstrom O., Stenhammar L., Ivarsson A. (2015). High adherence to a gluten-free diet in adolescents with screening-detected celiac disease. J. Pediatr. Gastroenterol. Nutr..

[83] Ferreira S., Chamorro M.E., Ortíz J., Carpinelli M.M., Giménez V., Langjahr P. (2018). Anti-transglutaminase antibody in adults with celiac disease and their relation to the presence and duration of gluten-free diet. Rev. Gastroenterol. Peru.

[84] Gładyś K., Dardzińska J., Guzek M., Adrych K., Małgorzewicz S. (2020). Celiac Dietary Adherence Test and Standardized Dietician Evaluation in Assessment of Adherence to a Gluten-Free Diet in Patients with Celiac Disease. Nutrients.

[85] Tursi A., Brandimarte G., Giorgetti G.M. (2003). Lack of usefulness of anti-transglutaminase antibodies in assessing histologic recovery after gluten-free diet in celiac disease. J. Clin. Gastroenterol..

[86] Burgin-Wolff A., Dahlbom I., Hadziselimovic F., Petersson C.J. (2002). Antibodies against human tissue transglutaminase and endomysium in diagnosing and monitoring coeliac disease. Scand. J. Gastroenterol..

[87] Vahedi K., Mascart F., Mary J.Y., Laberenne J.E., Bouhnik Y., Morin M.C., Ocmant A., Velly C., Colombel J.F., Matuchansky C. (2003). Reliability of antitransglutaminase antibodies as predictors of gluten-free diet compliance in adult celiac disease. Am. J. Gastroenterol..

[88] Matysiak-Budnik T., Malamut G., de Serre N.P., Grosdidier E., Seguier S., Brousse N., Caillat-Zucman S., Cerf-Bensussan N., Schmitz J., Cellier C. (2007). Long-term follow-up of 61 coeliac patients diagnosed in childhood: Evolution toward latency is possible on a normal diet. Gut.

[89] Kaukinen K., Sulkanen S., Maki M., Collin P. (2002). IgA-class transglutaminase antibodies in evaluating the efficacy of gluten-free diet in coeliac disease. Eur. J. Gastroenterol. Hepatol..

[90] Bufler P., Heilig G., Ossiander G., Freudenberg F., Grote V., Koletzko S. (2015). Diagnostic performance of three serologic tests in childhood celiac disease. Z. Gastroenterol..

[91] Bannister E.G., Cameron D.J., Ng J., Chow C.W., Oliver M.R., Alex G., Catto-Smith A.G., Heine R.G., Webb A., McGrath K. (2014). Can celiac serology alone be used as a marker of duodenal mucosal recovery in children with celiac disease on a gluten-free diet?. Am. J. Gastroenterol..

[92] Galli G., Carabotti M., Conti L., Scalamonti S., Annibale B., Lahner E. (2023). Comparison of Clinical, Biochemical and Histological Features between Adult Celiac Patients with High and Low Anti-Transglutaminase IgA Titer at Diagnosis and Follow-Up. Nutrients.

[93] Qureshi M.H. (2023). The Correlation Between Serum Anti-tissue Transglutaminase (Anti-tTG) Antibody Levels and Histological Severity of Celiac Disease in Adolescents and Adults: A Meta-Analysis. Cureus.

[94] Selby W.S., Painter D., Collins A., Faulkner-Hogg K.B., Loblay R.H. (1999). Persistent mucosal abnormalities in coeliac disease are not related to the ingestion of trace amounts of gluten. Scand. J. Gastroenterol..

[95] Sugai E., Nachman F., Vaquez H., Gonzalez A., Andrenacci P., Czech A., Niveloni S., Mazure R., Smecuol E., Cabanne A. (2010). Dynamics of celiac disease-specific serology after initiation of a gluten-free diet and use in the assessment of compliance with treatment. Dig. Liver Dis..

[96] Leffler D.A., Edwards George J.B., Dennis M., Cook E.F., Schuppan D., Kelly C.P. (2007). A prospective comparative study of five measures of gluten-free diet adherence in adults with coeliac disease. Aliment. Pharmacol. Ther..

[97] Leonard M.M., Weir D.C., DeGroote M., Mitchell P.D., Singh P., Silvester J.A., Leichtner A.M., Fasano A. (2017). Value of IgA tTG in Predicting Mucosal Recovery in Children with Celiac Disease on a Gluten-Free Diet. J. Pediatr. Gastroenterol. Nutr..

[98] Silvester J.A., Kurada S., Szwajcer A., Kelly C.P., Leffler D.A., Duerksen D.R. (2017). Tests for Serum Transglutaminase and Endomysial Antibodies Do Not Detect Most Patients with Celiac Disease and Persistent Villous Atrophy on Gluten-free Diets: A Meta-analysis. Gastroenterology.

[99] Rubio-Tapia A., Rahim M.W., See J.A., Lahr B.D., Wu T.T., Murray J.A. (2010). Mucosal recovery and mortality in adults with celiac disease after treatment with a gluten-free diet. Am. J. Gastroenterol..

[100] Nazario E., Lasa J., Schill A., Duarte B., Berardi D., Paz S., Muryan A., Zubiaurre I. (2022). IgA Deficiency Is Not Systematically Ruled Out in Patients Undergoing Celiac Disease Testing. Dig. Dis. Sci..

[101] Choung R.S., Khaleghi Rostamkolaei S., Ju J.M., Marietta E.V., Van Dyke C.T., Rajasekaran J.J., Jayaraman V., Wang T., Bei K., Rajasekaran K.E. (2019). Synthetic Neoepitopes of the Transglutaminase-Deamidated Gliadin Complex as Biomarkers for Diagnosing and Monitoring Celiac Disease. Gastroenterology.

[102] Ramírez-Sánchez A.D., Tan I.L., Gonera-de Jong B.C., Visschedijk M.C., Jonkers I., Withoff S. (2020). Molecular Biomarkers for Celiac Disease: Past, Present and Future. Int. J. Mol. Sci..

[103] Singh A., Pramanik A., Acharya P., Makharia G.K. (2019). Non-Invasive Biomarkers for Celiac Disease. J. Clin. Med..

[104] Ruiz-Carnicer Á., Garzón-Benavides M., Fombuena B., Segura V., García-Fernández F., Sobrino-Rodríguez S., Gómez-Izquierdo L., Montes-Cano M.A., Rodríguez-Herrera A., Millán R. (2020). Negative predictive value of the repeated absence of gluten immunogenic peptides in the urine of treated celiac patients in predicting mucosal healing: New proposals for follow-up in celiac disease. Am. J. Clin. Nutr..

[105] Bascunan K.A., Vespa M.C., Araya M. (2016). Celiac disease: Understanding the gluten-free diet. Eur. J. Nutr..

[106] Samasca G., Sur G., Lupan I., Deleanu D. (2014). Gluten-free diet and quality of life in celiac disease. Gastroenterol. Hepatol. Bed Bench.

[107] Balamtekin N., Aksoy C., Baysoy G., Uslu N., Demir H., Koksal G., Saltik-Temizel I.N., Ozen H., Gurakan F., Yuce A. (2015). Is compliance with gluten-free diet sufficient? Diet composition of celiac patients. Turk. J. Pediatr..

[108] Churruca I., Miranda J., Lasa A., Bustamante M.A., Larretxi I., Simon E. (2015). Analysis of Body Composition and Food Habits of Spanish Celiac Women. Nutrients.

[109] Kautto E., Ivarsson A., Norstrom F., Hogberg L., Carlsson A., Hornell A. (2014). Nutrient intake in adolescent girls and boys diagnosed with coeliac disease at an early age is mostly comparable to their non-coeliac contemporaries. J. Hum. Nutr. Diet..

[110] Wu J.H., Neal B., Trevena H., Crino M., Stuart-Smith W., Faulkner-Hogg K., Yu Louie J.C., Dunford E. (2015). Are gluten-free foods healthier than non-gluten-free foods? An evaluation of supermarket products in Australia. Br. J. Nutr..

[111] Shepherd S.J., Gibson P.R. (2013). Nutritional inadequacies of the gluten-free diet in both recently-diagnosed and long-term patients with coeliac disease. J. Hum. Nutr. Diet..

[112] Miranda J., Lasa A., Bustamante M.A., Churruca I., Simon E. (2014). Nutritional differences between a gluten-free diet and a diet containing equivalent products with gluten. Plant Foods Hum. Nutr..

[113] Vici G., Belli L., Biondi M., Polzonetti V. (2016). Gluten free diet and nutrient deficiencies: A review. Clin. Nutr..

[114] Martin J., Geisel T., Maresch C., Krieger K., Stein J. (2013). Inadequate nutrient intake in patients with celiac disease: Results from a German dietary survey. Digestion.

[115] MacCulloch K., Rashid M. (2014). Factors affecting adherence to a gluten-free diet in children with celiac disease. Paediatr. Child. Health.

[116] Burden M., Mooney P.D., Blanshard R.J., White W.L., Cambray-Deakin D.R., Sanders D.S. (2015). Cost and availability of gluten-free food in the UK: In store and online. Postgrad. Med. J..

[117] Missbach B., Schwingshackl L., Billmann A., Mystek A., Hickelsberger M., Bauer G., Konig J. (2015). Gluten-free food database: The nutritional quality and cost of packaged gluten-free foods. PeerJ.

[118] Leffler D., Schuppan D., Pallav K., Najarian R., Goldsmith J.D., Hansen J., Kabbani T., Dennis M., Kelly C.P. (2013). Kinetics of the histological, serological and symptomatic responses to gluten challenge in adults with coeliac disease. Gut.

[119] Pedoto D., Troncone R., Massitti M., Greco L., Auricchio R. (2020). Adherence to Gluten-Free Diet in Coeliac Paediatric Patients Assessed through a Questionnaire Positively Influences Growth and Quality of Life. Nutrients.

[120] Ludvigsson J.F., Leffler D.A., Bai J.C., Biagi F., Fasano A., Green P.H., Hadjivassiliou M., Kaukinen K., Kelly C.P., Leonard J.N. (2013). The Oslo definitions for coeliac disease and related terms. Gut.

[121] Elsahoryi N.A., Ibrahim M.O., Alhaj O.A. (2024). Adherence to the Gluten-Free Diet Role as a Mediating and Moderating of the Relationship between Food Insecurity and Health-Related Quality of Life in Adults with Celiac Disease: Cross-Sectional Study. Nutrients.

[122] Czaja-Bulsa G., Bulsa M. (2018). Adherence to Gluten-Free Diet in Children with Celiac Disease. Nutrients.

[123] Muhammad H., Reeves S., Ishaq S., Mayberry J., Jeanes Y.M. (2017). Adherence to a Gluten Free Diet Is Associated with Receiving Gluten Free Foods on Prescription and Understanding Food Labelling. Nutrients.

